# Hyperbaric oxygen therapy for brain abscesses: A useful adjuvant treatment for a faster recovery

**DOI:** 10.1007/s10143-025-03721-9

**Published:** 2025-07-30

**Authors:** A. Brunner, J. Lindenmann, K. Pistracher, A. Micko, A. Pichler, C. Enzinger, F. Smolle-Jüttner, S. Wolfsberger, S. Kurschel-Lackner

**Affiliations:** 1 Department of Neurosurgery, Medical University of Graz, Auenbruggerplatz 29, 8036 Graz, Austria; 2 Division of Thoracic and Hyperbaric Surgery, Department of Surgery, Medical University of Graz, Graz, Austria; 3 Department of Neurology, Medical University of Graz, Graz, Austria

**Keywords:** Brain abscess, Hyperbaric oxygen therapy, HBOT, Adjuvant therapy

## Abstract

Brain abscesses are still characterized by substantial case fatality rates and a high risk of permanent functional impairment. Standard treatment consists of long-term antimicrobial therapy and various neurosurgical interventions. In a few institutions, hyperbaric oxygen therapy (HBOT) is used as an additional treatment modality. The purpose of this study was to evaluate the effects of adjuvant HBOT on neurological and radiological outcomes in patients with brain abscesses. 55 patients with brain abscesses treated at the Medical University Clinic of Graz between 2004 and 2022 were included in this retrospective analysis. Thirty patients (54.5%) received standard therapy, consisting of long-term antimicrobial therapy and at least one neurosurgical intervention. Twenty-five patients (45.5%) additionally underwent HBOT. After three months, 24% of patients in the HBOT group and 10% in the non-HBOT group exhibited no residual abscess or pathological enhancement; at six-month follow-up, the percentage increased to 80% in the HBOT group compared to 46.7% in the non-HBOT group (p = 0.009). At 12-month follow-up, a symptom-free status (modified Rankin Scale 0) was attained by 60% of HBOT group patients and 30% of non-HBOT group patients (p = 0.046). The 12-month mortality rates for HBOT and non-HBOT groups were 12% (n = 3) and 20% (n = 6), respectively. No adverse effects related to HBOT were noted. Adjuvant HBOT significantly improved radiological outcome after 6 months, neurological outcome after 12 months and reduced mortality. HBOT may be taken into consideration in all patients with brain abscesses, particularly in cases with deep seated or multiple lesions and when antimicrobial and surgical treatment had failed.

## Introduction

To date, brain abscesses are still considered a serious, potentially life-threatening disease with high morbidity and mortality rates even in developed countries. Incidence rates of 0.3 to 1.3 per 100,000 per year have been reported in immunocompetent individuals, predominantly affecting males in their fourth decade of life [1–4]. Various risk factors are known for the development of brain abscesses, including an immunocompromised state, pre-existing infections, and trauma- or surgery-related cerebral pathogen exposure [1–7]. In the immunocompetent, approximately 95% of brain abscesses are caused by bacteria; in immunocompromised individuals they are more commonly derived from opportunistic pathogens and fungi [1, 2, 4, 5]. Most brain abscesses result from contiguous spread (40–50%) or haematogenous dissemination (30–40%) [5].

Brain abscesses develop in four stages, culminating in the formation of a mature collagen capsule typically observed after 14 days [8]. The clinical presentation is characterized by nonspecific symptoms, often manifesting as rapidly increasing signs of an intracranial mass and signs and symptoms of infection [1, 2, 5, 9]. Neuroimaging is crucial in the diagnostic workup of brain abscesses with postcontrast magnetic resonance imaging (MRI) regarded as the gold standard [5, 10]. Additional MRI sequences, such as diffusion-weighted imaging and apparent diffusion coefficient images, allow the differentiation of abscesses from neoplastic lesions [1, 2, 4, 5, 9–11]. Despite advancements, pathogen detection by blood cultures is successful in only 28%, while intraoperatively obtained specimens yield positive results in 68% of cases. Additional analysis of bacterial genetics by 16S ribosomal DNA sequencing can further increase the diagnostic yield [1, 2, 4, 5, 10, 12, 13].

Currently, there are still no evidence-based recommendations for the treatment of brain abscesses. The mainstay of treatment includes long-term antimicrobial therapy and neurosurgical interventions in most cases (87%). The purpose of surgery is to decrease abscess volume in order to improve the clinical condition and responsiveness to antimicrobial therapy, and to detect causative pathogens from obtained samples. Surgical techniques include aspiration of abscess contents via burr hole trepanation or microsurgical removal of the abscess formation via craniotomy [1, 2, 4, 5, 9–11, 14, 15].

Hyperbaric oxygen therapy (HBOT) is defined as breathing 100% oxygen under two to three-times the atmospheric pressure in a in a so-called hyperbaric chamber. According to the laws of Boyle–Mariotte, Dalton and Henry, the amount of oxygen dissolved in the plasma under hyperbaric conditions shows a considerable rise according to the pressure applied, resulting in a linear increase in arterial partial oxygen pressure (paO2). Under normobaric conditions, the paO2 ranges between 75 and 100 mmHg, whereas during HBOT, a paO2 between 1200 and 2000 mmHg is usually achieved. This HBOT‐induced high level of oxygen in the plasma and, in consequence, also in tissue, has several pharmacological effects which are used therapeutically for a variety of diseases [16]. The increased oxygen partial pressure during HBOT has bacteriostatic and bactericidal effects, increases the efficiency of antibiotic therapy by balancing hypoxia and acidosis, activates neutrophil-mediated phagocytosis, and promotes vasoconstriction and anti-inflammatory processes resulting in a decreased perifocal oedema and a more rapid abscess regression [17]. The Tenth European Consensus Conference on Hyperbaric Medicine recommended HBOT as an adjuvant therapy for patients with brain abscesses in which surgical interventions are contraindicated, in which conventional therapy has failed, in patients with multiple and/or deep-seated abscesses, or with abscesses in eloquent areas [18].

Up to now, there are few, exclusively retrospective, studies confirming a beneficial effect of adjuvant HBOT on the outcome of patients with brain abscesses [11, 15]. Although mortality has been reduced to 10–15% due to medical advances, morbidity is still up to 50% with persistent neurological deficits in 45% of affected patients. This study will focus on the outcome of patients with brain abscesses with adjuvant HBOT faced with a comparable group without HBOT in a single centre. To the best of our knowledge, this work to date represents the largest study on patients with brain abscesses receiving adjuvant HBOT.

## Material and Methods

Between January 2004 and December 2022, 63 patients with brain abscesses had been treated at the Department of Neurosurgery, University Hospital of Graz. After excluding patients younger than 18 years and patients with incomplete documentation, 55 patients remained for analysis.

### Diagnostics and surgical treatment

Diagnosis was confirmed by clinical symptoms (headache, fever, focal neurological deficits), imaging (cranial computed tomography (CT) or MRI of the brain), laboratory investigations (C-reactive protein, peripheral white blood cells, blood cultures), surgical exploration, and histological and microbiological evaluation of intraoperatively obtained specimens (aerobic and anaerobic bacteria, mycobacteria, fungi).

Diagnostic workup included analysis of predisposing conditions and detection of infectious sources.

All patients underwent standard treatment of brain abscesses (antimicrobial therapy, source control and surgical treatment, if indicated). An empirical antibacterial treatment was initiated as soon as possible, starting immediately after blood cultures have been sampled. Antimicrobial therapy was modified according to detected pathogens and antibiogram.

Surgical treatment consisted of needle aspiration via burr hole trepanation or abscess evacuation and resection of varying extent by craniotomy. The type of surgical procedure was based on patient’s general condition and brain abscess characteristics (number, maturity/capsule formation, size, location).

The first follow-up MRI was performed on the first postoperative day. In critically ill patients requiring intensive care, it was based on their condition, but was usually done within the first week after surgery.

### Hyperbaric Oxygen treatment (HBOT)

Decision for adjuvant HBOT was made individually upon an interdisciplinary consensus, based on the patient’s general condition (fitness for surgery, transportability) and abscess characteristics (size, number, localization). Prior to HBOT, each patient had a chest x-ray and underwent an otorhinolaryngological examination to confirm the ability for pressure equalization in the middle ear. If required, a myringotomy was performed or a tympanostomy was inserted.

HBOT was administered in cooperation with the Division of Thoracic and Hyperbaric Surgery in a large walk-in drive-in hyperbaric chamber located in the surgical building complex. The facility allows the treatment of outpatient as well as compromised in-hospital cases. It provides complex non-invasive and invasive monitoring during hands-on treatment of critically ill patients, who are transferred into the chamber in their respective beds, obviating the need for manipulation and for disconnecting of lines.

Treatment was given once daily at a pressure of 2.2 atmospheres absolute (ATA). The compression rate was 0.1 bar/min equalling 10 min until reaching treatment pressure. Patients remained at 2.2 ATA for 60 min with one oxygen break of 10 min. The chamber was decompressed within 10 min including a 5-min stop at 3 m. During HBOT patients breathed 100% oxygen via tightly fitting masks, endotracheal tube or tracheostomy. A total of 30 treatment sessions was scheduled, but the number of treatment sessions applied varied depending on the clinical course.

Data were collected retrospectively including neurological condition on admission, predisposing conditions, source and route of infection, abscess localisation and volume, type of neurosurgical intervention, causative pathogens, duration of antimicrobial treatment, radiological and neurological outcome, and rate of complications.

### Outcome

Follow-ups were performed three, six, and twelve months after initial diagnosis including a neurological (including the modified Rankin Scale [mRS]) and radiological examinations (MRI of the neurocranium, including contrast-enhanced sequences and calculation of abscess volume). Brain abscesses and residual contrast-enhancing lesions, were segmented based on contrast-enhanced MRI using the Medtronic StealthStation S8 Neuronavigation System to determine their volume.

### Statistical analysis

Data were entered into a computerized database and analysed. Age is reported as median with range (minimum–maximum), categorical data are displayed as frequencies with percentages in parentheses.

To determine the statistical significance of group differences, we used the Student T-Test for independent samples for age and the Mann–Whitney-U test for ordinal parameters. The relationship between categorical parameters is presented in contingency tables and analysed with Pearson’s chi-squared test. In either case, we determined exact p-values; we considered p ≤ 0.05 to be statistically significant. Computations were performed using the statistical package IBM SPSS Statistics Version 28 (Release 28.0.0.0, 2022. Armonk, NY, USA: International Business Machines Corporation).

## Results

### Demographic characteristics (Table 1)

**Table 1:** Demographic characteristics

*Parameter*	*HBO*		*non HBO*		*p*
	*n*	*(%)*	*n*	*(%)*	
	25	(40%)	30	(60%)	
Age at diagnosis (median)	52		51,5		
Gender					0,386
male	19	(76%)	19	(63,3%)	
female	6	(24%)	11	(36,7%)	
Underlying diesase/predisposing factor					0,952
cryptogenic	3	(12%)	2	(6,7%)	
dental infection	8	(32%)	10	(33,3%)	
malignant disease	1	(4%)	1	(3,3%)	
tonsillitis	1	(4%)	1	(3,3%)	
mykosis	1	(4%)	1	(3,3%)	
haemato-oncological disease	1	(4%)	1	(3,3%)	
hereditary hemorrhagic teleangiectasia	1	(4%)	0	(0%)	
sinusitis	3	(12%)	4	(13,3%)	
posttraumatic	2	(8%)	1	(3,3%)	
otitis, mastoiditis	2	(8%)	2	(6,7%)	
pneumonia	1	(4%)	1	(3,3%)	
bacteremia/sepsis	0	(0%)	1	(3,3%)	
immunocompromised state	0	(0%)	3	(10%)	
other cause	1	(4%)	2	(6,7%)	
Source of infection					0,636
disseminated	8	(32%)	7	(23,3%)	
haematogenous	12	(48%)	14	(46,7%)	
immunogen	3	(12%)	6	(20%)	
not determined	2	(8%)	3	(10%)	
Charlson Comorbidity Index					0,197
0	6	(24%)	6	(20%)	
1	4	(16%)	5	(16,7%)	
2	1	(4%)	8	(26,7%)	
3	7	(28%)	4	(13,3%)	
4	5	(20%)	2	(6,7%)	
5	1	(4%)	3	(10%)	
6	1	(4%)	2	(6,7%)	
5-Factor Modified Frailty Index					0,634
0	5	(20%)	10	(33,3%)	
1	11	(44%)	10	(33,3%)	
2	6	(24%)	7	(23,3%)	
3	3	(12%)	2	(6,7%)	
4	0	(0%)	1	(3,3%)	
5	0	(0%)	0	(0%)	
mRS at admission					0,841
no significant disability (1)	1	(4%)	5	(16,7%)	
slight disability (2)	8	(32%)	5	(16,7%)	
moderate disability (3)	2	(8%)	4	(13,3%)	
moderately severe disability (4)	8	(32%)	8	(26,7%)	
severe disability (5)	6	(24%)	8	(26,7%)	
GCS at admission					0,213
mild impairment or alert (13–15)	20	(80%)	20	(66,7%)	
moderate impairment (9–12)	4	(16%)	5	(16,7%)	
severe impairment (3–8)	1	(4%)	5	(16,7%)	

Abbreviations: *n* number of patients, *mRS* modified Rankin Scale, *GCS* Glasgow Coma Score

Fifty-five patients with brain abscesses met the inclusion criteria. The mean age at diagnosis was 53 years (range 22–79 years) and the male-to-female ratio was 2:1. Depending on whether patients underwent adjuvant HBOT or not, they were placed into group HBOT (n = 25 cases) or group non-HBOT (n = 30 cases). The baseline demographic and clinical features were not significantly different between the two groups (Table 1).

The most common sources of infection were dental (n = 18), followed by otolaryngological (n = 13), and posttraumatic and immunodeficiency (n = 3). In 15 patients, brain abscesses were caused by contiguous spread, in 26 patients by haematogenous spread; the remaining 14 cases were categorized as cryptogenic.

Comorbidities, assessed using the Charlson Comorbidity Index (CCI) and the 5-factor modified Frailty Index (FI-5), revealed a relatively high burden of comorbidities. Twelve patients (21.8%) had no comorbidities (CCI 0), while nine patients (16.4%) had a CCI of 1. Regarding frailty, 15 patients (27.3%) had an FI-5 score of 0, indicating no frailty, whereas five patients (9.1%) had an FI-5 score of 3 or higher.

At admission, patients most frequently presented a moderate to severe disability (mRS 4, n = 16; 29.1%) or severe disability (mRS 5, n = 14; 25.5%). Twenty-nine patients (52.7%) developed abscess-associated symptoms one month after the onset of an infectious disease or another triggering event.

### Characteristics of brain abscesses (Table 2)

**Table 2:** Characteristics of brain abscesses

*Parameter*	*HBO*		*non HBO*		*p*
	*n*	*(%)*	*n*	*(%)*	
Abscess localization					0,442
frontal	12	(48%)	8	(26,7%)	
temporal	3	(12%)	7	(23,3%)	
parietal	3	(12%)	3	(10%)	
occipital	4	(16%)	2	(6,7%)	
frontotemporal	0	(0%)	2	(6,7%)	
frontoparietal	0	(0%)	1	(3,3%)	
parietooccipital	0	(0%)	1	(3,3%)	
diencephal	0	(0%)	1	(3,3%)	
cerebellar	1	(4%)	0	(0%)	
multiple	2	(8%)	5	(16,7%)	
Abscess volume (cm3)					0,581
1–10	5	(20%)	2	(6,7%)	
11–20	4	(16%)	6	(20%)	
21–30	5	(20%)	8	(26,7%)	
31–40	2	(8%)	3	(10%)	
41–50	2	(8%)	3	(10%)	
51–60	3	(12%)	2	(6,7%)	
not determined	4	(16%)	6	(20%)	
Abscess distance from brain surface (mm)					0,602
1–5	16	(64%)	19	(63,3%)	
6–10	2	(8%)	5	(16,7%)	
11–15	4	(16%)	2	(6,7%)	
16–20	0	(0%)	1	(3,3%)	
21–25	0	(0%)	0	(0%)	
26–30	1	(4%)	0	(0%)	
31–35	0	(0%)	0	(0%)	
36–40	0	(0%)	0	(0%)	
41–45	0	(0%)	1	(3,3%)	
not determined	2	(8%)	2	(6,7%)	

Abbreviations: *n* number of patients, *cm*^*3*^ cubic centimeters, *mm* millimeters

Abscesses were most frequently located in the frontal lobes (n = 20; 36.4%), followed by the temporal lobes in 10 (18.2%), and 6 (10.9%) in the occipital and parietal lobes, respectively. Four abscess formations (7.3%) affected more than one lobe (n = 2; 3.6% frontotemporal, n = 1; 1.8% frontoparietal, n = 1 1.8% parietooccipital). Posterior fossa (n = 1; 1.8%) and diencephalon (n = 1, 1.8%) were rare locations. Multiple abscesses were present in 7 cases (12.7%).

At diagnosis, most abscesses (n = 13; 23.6%) had a median volume of 25.7 cm3 (interquartile range, 14.5–38.5).

Regarding abscess depth, most were located superficially: 35 patients (63.6%) had abscesses within 1–5 mm of the brain surface, while deeper abscesses (6 mm or more) were less common (n = 10; 18.2%).

There were no statistically significant differences in these parameters between the two treatment groups (HBOT, non-HBOT).

There were no statistically significant differences in these parameters between the two treatment groups (HBOT, non-HBOT.

### Microbiological analysis (Table 3)

**Table 3:** Microbiological analysis

*Parameter*	*HBOT*		*non HBOT*		*p*
	*n*	*(%)*	*n*	*(%)*	
Microbiological analysis					0,564
Streptococcus intermedius	4	(16,0%)	10	(33,3%)	
Staphylococcus aureus	1	(4%)	1	(3,3%)	
Aspergillus fumigatus	2	(8%)	0	(0%)	
Streptococcus constellatus	1	(4%)	1	(3,3%)	
Streptococcus pneumoniae	0	(0%)	1	(3,3%)	
sterile	7	(28%)	6	(20%)	
not recorded	2	(8%)	1	(3,3%)	

Abbrevations:* n *number of patients

Pathogen detection in blood cultures or in intraoperatively obtained specimens was successful in 39 patients (70.9%). The most frequently cultured microorganisms were Streptococcus intermedius (n = 14; 25.5%), Staphylococcus aureus (n = 2; 3.6%), Aspergillus fumigatus (n = 2; 3.6%), Streptococcus constellatus (n = 2; 3.6%), and Streptococcus pneumoniae (n = 1; 1.8%).

There were no statistically significant differences in these parameter between the two treatment groups (HBOT, non-HBOT.

### Surgical treatment

All patients underwent surgery. In 34 cases (61.8%), a craniotomy with abscess evacuation and resection of varying extent was performed. Aspiration via burr hole trepanation was done in 17 cases (30.9%). In four patients (7.3%), an external ventricular drainage had to be inserted due to hydrocephalus with ventriculitis.

The abscess capsule was completely excised in 13 patients (23.6%), partially excised in 17 patients (30.9%), and not excised in 25 patients (45.5%). The latter group mostly includes patients who underwent burr hole aspiration or insertion of an external ventricular drainage.

There were no statistically significant differences between the groups.

#### HBOT

Twenty-five patients (45.5%) underwent adjuvant HBOT. Most patients (n = 11, 44%) were treated for 29–35 days. Treatment durations varied for the other patients: 8–14 days in five patients (20%), 22–28 days for four patients (16%), 1–7 days in four patients (16%), and 15–21 days in one patient (4%).

Despite a previous otorhinolaryngological exanimation and intervention, one patient (4%) had to discontinue treatment due to problems with pressure equalization. No adverse events related to HBOT have been observed.

### Outcome

Patients in the HBOT group had a significantly longer total hospital stay (mean = 29.92 days) compared to the non-HBOT group (mean = 23.23 days; p = 0.010) and a prolonged stay on regular ward (mean = 22.36 days vs. 12.66 days; p = 0.019). There were no statistically significant differences between the two groups regarding ICU stay duration (mean = 6.27 days for HBOT vs. 10.10 days for non-HBOT; p = 0.376) and the duration of mechanical ventilation (mean = 3.38 days in both groups; p = 0.553).

### Radiological outcome (Table 4)

**Table 4:** Radiological Outcome

*Parameter*	*HBO*		*non HBO*		*p*
	*n*	*(%)*	*n*	*(%)*	
Abscess volume (cm^3^ postoperatively)					0,097
0	0	(0%)	0	0%)	
1–10	12	(24%)	20	(66,7%)	
11–20	2	(8%)	4	(13,3%)	
21–30	4	(16%)	1	(3,3%)	
31–40	1	(4%)	0	(0%)	
41–50	1	(4%)	2	(6,7%)	
51–60	0	(0%)	0	(0%)	
61–70	1	(4%)	0	(0%)	
71–80	0	(0%)	0	(0%)	
81–90	1	(4%)	0	(16%)	
not recorded	3	(12%)	3	(10%)	
Abscess volume (cm^3^ after 3 months)					0,08
0	6	(24%)	3	(10%)	
1–10	13	(52%)	17	(56,7%)	
11–20	1	(4%)	3	(0%)	
21–30	1	(4%)	0	(0%)	
31–40	0	(0%)	0	(0%)	
41–50	0	(0%)	1	(3,3%)	
51–60	0	(0%)	2	(6,7%)	
not recorded	4	(16%)	4	(13,3%)	
Abscess volume (cm^3^ after 6 months)					**0,009**
0	20	(80%)	14	(46,7%)	
1–10	0	(0%)	6	(20%)	
11–20	0	(0%)	0	(0%)	
21–30	0	(0%)	0	(0%)	
31–40	0	(0%)	0	(0%)	
41–50	0	(0%)	0	(0%)	
51–60	0	(0%)	0	(0%)	
not recorded	5	(20%)	10	(33,3%)	
Abscess volume (cm3 after 12 months)					0,477
0	20	(80%)	17	(56%)	
1–10	1	(4%)	1	(3,3%)	
11–20	0	(0%)	1	(3,3%)	
21–30	0	(0%)	0	(0%)	
31–40	0	(0%)	0	(0%)	
41–50	0	(0%)	0	(0%)	
51–60	0	(0%)	0	(0%)	
not recorded	4	(16%)	11	(36,7%)	

Abbreviations: *n* number of patients, *cm*^*3*^ cubic centimeters

No significant difference in postoperative abscess volume was observed between the two groups (p = 0.097). However, a significant correlation was found between the postoperative abscess volume and the extent of abscess capsule excision (p = 0.027), with the volume being significantly smaller in cases of complete excision.

After 3 months, no residual abscess (no pathological enhancement) was detectable in 6 patients (24%) from the HBOT group and in 3 patients (10%) from the non-HBOT group (p = 0.08).

At 6-months follow-up, there was no evidence of residual abscess (no pathological contrast enhancement) in 20 patients (80%) from the HBOT group, as opposed to 14 patients (46.7%) in the non-HBOT group (p = 0.009).

Differences in terms of decreased contrast enhancement and reduction of abscess volume were clearly visible after 3 months in favour of the HBOT group (p = 0.08), reaching statistical significance at the 6-month interval (p = 0.009). Finally, after 12 months, the outcomes between the two cohorts demonstrated comparability (p = 0.477). In the HBOT group, 20 patients (80%) showed complete resolution of abscesses, compared to 17 patients (56%) in the non-HBOT group. Small residual abscess volumes between 1 and 10 cm^3^ were recorded in 1 patient (4%) in the HBOT group and in 1 patient (3.3%) in the non-HBOT group. Abscess volumes greater than 10 cm^3^ (specifically 11–20 cm^3^) were noted in 1 patient (3.3%) in the non-HBO group, with no cases in the HBOT group.

**Fig. 1:**
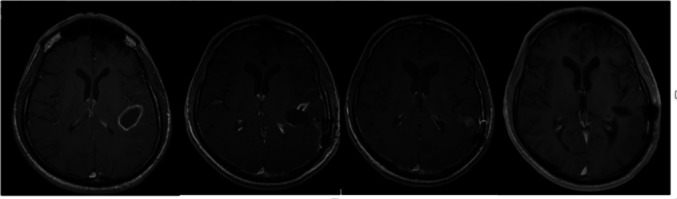
Sequential axial post-contrast T1- weighted MRI images obtained in a 72-year-old patient with right frontal brain abscess. A: Detection of a large abscess formation with intraventricular rupture, B: MRI performed on postoperative-day 1 after craniotomy, abscess evacuation and partial resection, C: MRI performed 20 days after initiation of HBOT showing further remission, D: MRI at 6-weeks follow up demonstrating no residual abscess

**Fig. 2:**
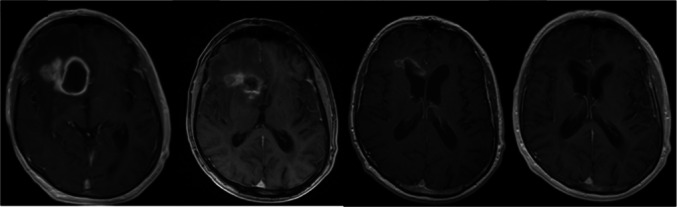
Sequential axial post-contrast T1- weighted MRI images obtained in a 49-year-old patient with left temporal brain abscess. A: Detection of the abscess formation, B: MRI performed on postoperative-day 1 after craniotomy, abscess evacuation and partial resection, C: MRI performed 28 days after initiation of HBOT showing further remission, D: MRI at 3-month follow up demonstrating no residual abscess

All measurements were based on contrast-enhanced MRI examinations (Figs. 1 and 2).

### Neurological outcome (Table 5, Fig. 3)

At the 12-month follow-up, a symptom-free status (mRS 0) was observed in 15 (60%) patients within the HBOT group and 9 (30%) patients within the non-HBOT group (p = 0.046). The 12-month mortality rates for the HBOT and non-HBOT groups were 12% (n = 3) and 20% (n = 6), respectively.

**Table 5:** Neurological outcome

*Parameter*	*HBOT*		*non HBOT*		*p*
	*n*	*(%)*	*n*	*(%)*	
Neurological Outcome (mRS after 12 months)					**0,046**
no symptoms (0)	15	(60%)	9	(30%)	
no significant disability (1)	4	(16%)	8	(26,7%)	
slight disability (2)	0	(0%)	5	(16,7%)	
moderate disability (3)	2	(8%)	1	(3,3%)	
moderately severe disability (4)	0	(0%)	0	(0%)	
severe disability (5)	0	(0%)	0	(0%)	
dead (6)	3	(12%)	6	(20%)	
not recorded	1	(4%)	1	(3,3%)	

Abbreviations: *n* number of patients, *mRS* modified Rankin Scale

**Fig. 3:**
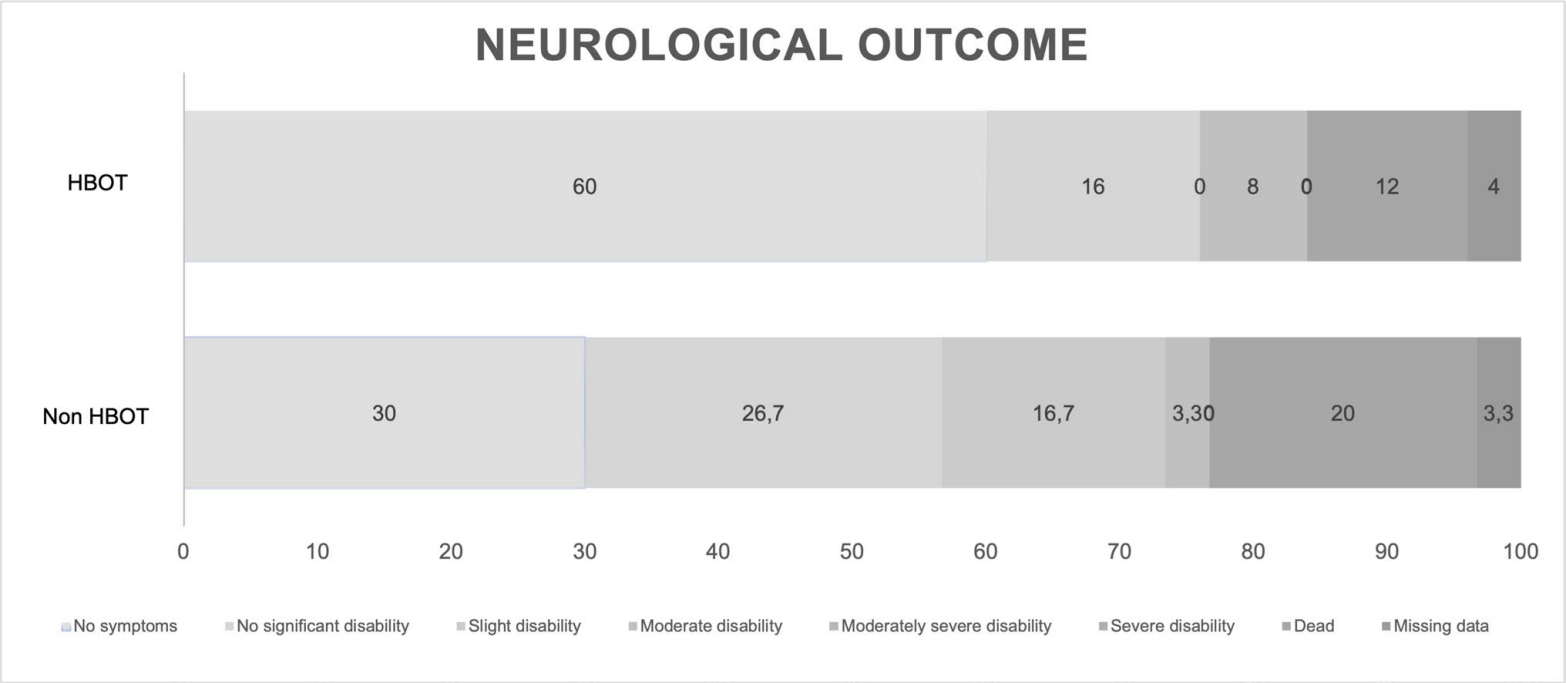
Neurological outcome

## Discussion

Despite medical progress over the last decades, brain abscesses are still associated with a high risk of permanent functional impairment, a number of complications, and can be life-threatening. However, evidence-based treatment recommendations are lacking. Conventional therapy, consisting of long-term antimicrobial therapy and neurosurgical interventions may fail; this can be explained by poor penetration of antimicrobial agents across the blood–brain barrier as well as into the abscess. In addition, the hypoxic and acidotic environment prevailing in abscesses leads to decreased efficacy of antimicrobial agents. The decreased oxygen level in abscesses impedes immune cell function; for example, the phagocytosis capacity of leukocytes is inhibited [17].

### HBOT Mechanisms

Since the 1980s, HBOT has been used as an adjuvant treatment option for patients with brain abscesses. In those early years, it had been found that HBOT causes considerable reduction of cerebral oedema and intracranial pressure via cerebral vasoconstriction. Despite vasoconstriction, hyperoxygenation secures abundant oxygen supply to the brain, which is supported by improved blood rheology [19]. Moreover, HBOT exerts direct toxicity on bacteria. It enhances the bactericidal capacity of leukocytes, and redresses the acidotic environment in abscesses, thus increasing the effects of antibiotics and shortening the duration of antimicrobial therapy [17, 20].

Though a neuroprotective impact of HBOT had been documented for many years, only recent studies are elucidating the different underlying molecular mechanisms of HBOT-effects in neural tissue. HBOT stabilizes hypoxia induced factor 1 (HIF-1), which reduces the release of inflammatory cytokines by macrophages and neutrophils [21]. Further mechanisms with relevance for neurologic disorders are the downregulation of matrix metalloproteinase-2 (MMP-2), MMP-9 and interleukin (IL-6) which, in an animal experiment of spinal cord injury (SCI), inversely correlated with the spinal water content, thus promoting recovery [22]. In another experimental study of SCI, injury-induced increased levels of p-mTOR, which regulates stem-cell maintenance, growth but also autophagy, as well as inflammatory cytokines and apoptosis in the spinal cord were attenuated [23]. Experimental neuronal trauma causes increase of the ratio of anti-apoptotic Bcl-2 to pro-apoptotic Bcl-2-associated X protein (BAX) which determines the survival or death of cells following an apoptotic stimulus. HBOT rapidly restored the balance of Bcl-2 and BAX, thereby inhibiting cell death and facilitating neuronal recovery [24]. Also, by modifying proteinkinase B and beta-catenin, HBO was able to attenuate apoptosis following experimental craniocerebral trauma. [25]. In 2022, Xia et al. studied the influence of HBOT on apoptosis following experimental traumatic brain injury. HBOT downregulated pro-inflammatory kinases and chemoattractants as well as nuclear factor Kappa-B, a transcription factor activated during stress response, resulting in reduction of neuronal apoptosis and functional improvement [26].

### Studies on HBOT for Brain Abscesses

Despite the plethora of beneficial experimental findings, undisputed clinical evidence based on well-designed studies is still lacking. The Tenth European Consensus Conference on Hyperbaric Medicine recommended the integration of HBOT in the treatment of intracranial abscesses in the presence of multiple abscesses, abscesses in deep or dominant locations, patients in poor general condition, contraindications to surgery, or failure of conventional treatment measures (type 1 recommendation with level C evidence, due to the paucity of studies) [27].

Previous studies have demonstrated the beneficial effects of adjuvant HBOT on abscess recurrence and clinical outcome. Bartek et al. reported a significantly better treatment response and long-term outcome of twenty patients with brain abscesses treated with adjuvant HBOT compared to a conventionally treated control group [15]. Few case series suggest beneficial effects of adjuvant HBOT in the treatment of brain abscesses. Kurschel et al. reported a very favourable radiological and functional outcome in a paediatric cohort of 5 children with brain abscesses, a finding that was corroborated by Lackner et al. in 2009 and by Maritschnegg et al. in 2011 [11, 28, 29]. Another case series by Kutlay et al. indicates a better infection control with shortened length of antibiotic therapy in an adult cohort of 13 patients [30].

### Current Study Results

In our retrospective study, we evaluated the effect of adjuvant HBOT on radiological and functional outcome of patients with brain abscesses. The patient cohort consisted of 55 patients treated between 2004 and 2022; 30 patients (54.5%) received standard therapy (long-term antimicrobial therapy and at least one neurosurgical intervention), whereas 25 patients (45.5%) additionally underwent HBOT. The decision regarding adjuvant HBOT was made individually on an interdisciplinary consensus. To our knowledge, this study reports on the largest series of patients to date with brain abscesses undergoing adjuvant HBOT. Although there was no blinded randomisation to treatment groups, as this has been no prospectively designed study but dictated by clinical needs and best practice, it needs to be particularly emphasized that both groups, HBOT-treated and non-HBOT-treated, are comparable in group size, demographic data, abscess characteristics, and neurosurgical interventions, with no statistically significant differences or major imbalances. This distinguishes our report from the comparative cohort study on this topic by Bartek et al. where cohorts were smaller and more heterogeneous, lacking detailed information on comorbidities. This data may therefore suggest that patients in poorer clinical condition were more likely to be allocated to the non-HBOT group [15]. Our study shows in line with previous findings a male predominance (male-to-female ratio = 2:1) [1, 2, 5, 31]. In contrast to earlier findings, however, the median age at first presentation was about 20 years higher in our cohort [1, 2, 5, 31]. This result might be explained by an increasing life expectancy and by the overall medical progress over the last decades; as a result, previous studies covering time periods from the last century may not be fully comparable [1]. Consistent with the literature, 92% of our patients presented with a focal neurological deficit and 22% showed a severly impaired consciousness at admission [1, 4, 5, 9, 31]. In our study population, almost one third of brain abscesses originated from dental infections (32.7%). This is reflected by the most frequently identified odontogenic bacteria (Streptococcus intermedius, Streptococcus constellatus). The detection of mycosis was clearly higher in our study (3.6%), than described in the literature (1%) [1]. This might be explained by the larger proportion of immunocompromised patients. Most brain abscesses were located in the frontal and temporal lobe and multiple abscess formations were found in 12.7% (consistent with 18% in the literature). Overall, our patient characteristics are in line with previous reports [1, 4, 31]. All patients underwent surgery, therefore, the percentage of surgically treated patients was higher than in previous studies (87–92%) [1, 14, 31]. Most likely, this refers the natural selection bias of including patients treated at a neurosurgical department. Craniotomy for abscess evacuation and resection by varying degrees was performed most frequently (61.8%), whereas abscess aspiration via burr hole was done in a considerably lower proportion (30.9%; previous studies: 25% resection vs. 66% aspiration) [1, 14, 31]. In both cases with detection of Aspergillus fumigatus, a craniotomy and extirpation of the lesions was performed. This inconsistency in our study population might be explained first by the substantial abscess volumes (in 27.3% of cases the abscess volume exceeded 31 cm^3^) and the favourable location of most abscesses (frontal, temporal). Second, this could be attributed to a selected patient population, mainly referred to our department when surgery was indicated either for volume reduction and/or pathogen identification.

No HBOT-related adverse events occurred. This is consistent with the findings of other authors ^[[11,15,30]]^^.^ Our 4% rate of discontinuation of HBOT is also in accordance with other studies which reported an unforeseen stop of treatment in less than 10% [11]. Overall, HBOT is regarded as a safe and low-complication treatment.

Radiological and neurological outcomes were evaluated three, six, and twelve months after admission. The current study found a statistically significant better outcome in the HBOT group for both outcomes. After 3 months, radiologic characteristics such as contrast enhancement and abscess volume were clearly less pronounced in the HBOT group than in the non-HBOT group. At 6-month follow-up, this difference showed statistical significance. The radiologic outcomes equalized between the two cohorts after one year.

Neurologically at 12-month follow-up, 60% of patients in the HBOT group achieved a symptom-free status (mRS 0), in contrast to 30% in the non-HBOT group. The 12-month mortality rates for the HBOT and non-HBOT groups were 12% (3 cases) and 20% (6 cases), respectively.

The outcome in the non-HBOT cohort is consistent with data from a previous systematic review and a meta-analysis by Brower et al. including 123 studies and 9699 patients [1]. The mortality in our HBOT group was lower when compared to the mortality rate in this review (12% vs. 22%) [1]. Bartek et al. reported a remarkably low mortality rate of 0% in his study group (both HBOT and non-HBOT), however, this result does not support the previous research. The 12-month mortality rate in our series (16.4%) might be attributed again to a relatively high proportion of patients in an immunocompromised state or with malignant haematological disease (9%), most likely reflecting the patient selection of a tertiary academic referral centre.

## Summary

The present study increases the number of reported HBOT in patients with brain abscesses from 50 to 75, thereby significantly adding to the literature. Considering a statistically significant better outcome and the absence of adverse effects, it could be reasonable to extend the indication guidelines for the use of HBOT in brain abscesses. Cases with surgically limited options, multiple lesions, and failure of antimicrobial and surgical treatment are of particular interest for an adjuvant HBOT; however, based on the results, HBOT could be taken into consideration in all patients with brain abscesses for a faster recovery. Further prospective, randomized studies which larger patient groups are warranted to generate sufficient data for the development of clear treatment guidelines of HBOT in brain abscesses.

## Limitations

Limitations are given due to the retrospective study design; the study carries the risk of detection and selection biases. A further limitation of our study is the lack of a standardized follow-up.

A limited availability of hyperbaric chambers may potentially restrict the widespread application of HBOT interventions.

Despite presenting the largest study on this topic to date, a limitation of this study is the relatively small sample size, which may affect the generalizability of the findings. Larger multicentre studies with more extensive cohorts are needed to validate these results.

## Conclusion

According to our data, adjuvant HBOT is a safe adjuvant treatment option for patients with brain abscess and has the potential to improve radiological and neurological outcome of this life-threatening disease. Therefore, HBOT should be taken into consideration in all patients with brain abscesses, particularly in cases with deeply located or multiple lesions and when antimicrobial and surgical treatment had failed.

## Data Availability

No datasets were generated or analysed during the current study.
